# Supplementary material for: Convergent neural signatures of speech prediction error are a biological marker for spoken word recognition
Source: Nat Commun. 2024 Nov 18;15:9984. doi: 10.1038/s41467-024-53782-5 (PMC11574182; doi:10.1038/s41467-024-53782-5)
Supplement: Supplementary file 1 — Supplementary Information [file 41467_2024_53782_MOESM1_ESM.pdf]

Supplementary information

Stimulus properties

A summary of stimulus properties is shown in Figure S4A and S4B. Based on frequency counts, and phonological transcriptions in CELEX, the average conditional probability of syllable 2 given syllable 1 (p(Syl2|Syl1)) for Strong prediction items was 0.574 (SD = 0.332) while for Weak items it was 0.019 (SD = 0.029). Strong items were also associated with smaller syllable entropy (calculated as the negative sum of log-transformed p(Syl2|Syl1) over all possible syllables; mean = 1.34, SD = 1.11) than Weak items (mean = 2.99, SD = 0.99). Strong and Weak items had similar log frequencies (Strong: mean = 4.90, SD = 1.56; Weak: mean = 3.89, SD = 1.62) although the difference was significant (independent samples $t(62) = 2.53$, $p = 0.014$, $d = 0.632$, $CI_{95\%}$ = [0.211, 1.802]). However, all reported MEG and fMRI effects involving correlations with conditional probabilities (Figures 2B, 3B and 4D) survive when controlling for differences in word frequency (using partial Spearman correlations). Moreover, p(Syl2|Syl1) is the strongest predictor of listeners' behaviour in a free report gating task (conducted in a separate group of listeners; see Figure S1E and SI Methods for more details), as compared with either word frequency or syllable entropy (see web-based gating study results reported below). Thus, we are confident that our effects of prediction strength after Syl2 onset stem from differences in conditional probability rather than differences in word frequency.

For the fMRI experiment (Figure S4A), the Syl1 portion of Strong items was lower in acoustic intensity (independent samples $t(62) = -2.14$, $p = 0.036$, $d = -0.535$, $CI_{95\%}$ = [-0.550, -0.019]) and longer in duration (independent samples $t(62) = 3.47$, $p < .001$, $d = 0.868$, $CI_{95\%}$ = [-26.1, 97.1]) than Weak items. The RMS intensity for Syl1 and Syl2 was separately normalised over items for the MEG experiment (which was conducted after the fMRI experiment) and did not differ between conditions (Figure S4B). All other stimulus properties did not differ between the fMRI and MEG experiments. Importantly, there were no acoustic differences between conditions for the Syl2 portion of our stimuli which were acoustically identical in all conditions. Neural responses to Syl2 are the key focus of this study since it is representations of Syl2 that distinguish sharpened signal and prediction error accounts.

Cued recall task

After the fMRI scans, we assessed listeners' memory for the pseudowords in a cued recall task (see SI Methods). Recall accuracy (percentage correct) was around 10% on average over participants, with most participants achieving 5% or higher (see Figure S1D). These data indicate the recall task was difficult. Importantly however, they also suggest that participants could perform the task to some degree and hence were attentively processing the pseudoword stimuli. Recall accuracy was better for pseudowords formed with Strongly- versus Weakly-predicting first syllables (one-tailed $t(16) = 1.768$, $p = .048$, $d = 0.429$, $CI_{95\%} = [-.549, 6.064]$), consistent with the proposal that items evoking strong prediction errors are learned more readily than items evoking weak prediction errors [see Discussion].

Web-based gating study

Listeners' reports of the identity of spoken words with second syllables replaced by noise (Strong+Noise and Weak+Noise stimuli, see SI Methods) were combined over participants (N=110) to generate the proportion of responses that correspond to the target words. For these items, p(Syl2|Syl1) was strongly correlated with the proportion of listeners' responses matching the target word ($rho(62) = .636$, $p < .001$, Fisher's $z = 0.748$, $CI_{95\%} = [.460, .761]$; see Figure S1E) and this effect remains significant when other lexical predictors (entropy and word frequency) are factored out ($rho(62) = 0.341$, $p < 0.01$, Fisher's $z = 0.748$, $CI_{95\%} = [.460, .761]$). This finding shows that more strongly predicted second syllables were more likely to be reported by listeners than weakly predicted syllables when replaced with noise. Moreover, while both entropy and word frequency also correlate with gating responses ($rho(62) = -0.513$, $p < .001$, Fisher's $z = -0.567$, $CI_{95\%} = [-.674, -.306]$; and $rho(62) = 0.259$, $p < .05$, Fisher's $z = 0.265$, $CI_{95\%} = [.014, .474]$), neither of these factors predict gating responses when p(Syl2|Syl1) is factored out (both $p > .05$, see Figure S1E). These findings confirm that our experimental manipulation of prediction strength was successful, and that measures of prediction strength based on conditional probability computed from lexical statistics are an accurate predictor of listening behaviour.

Supplementary information figures

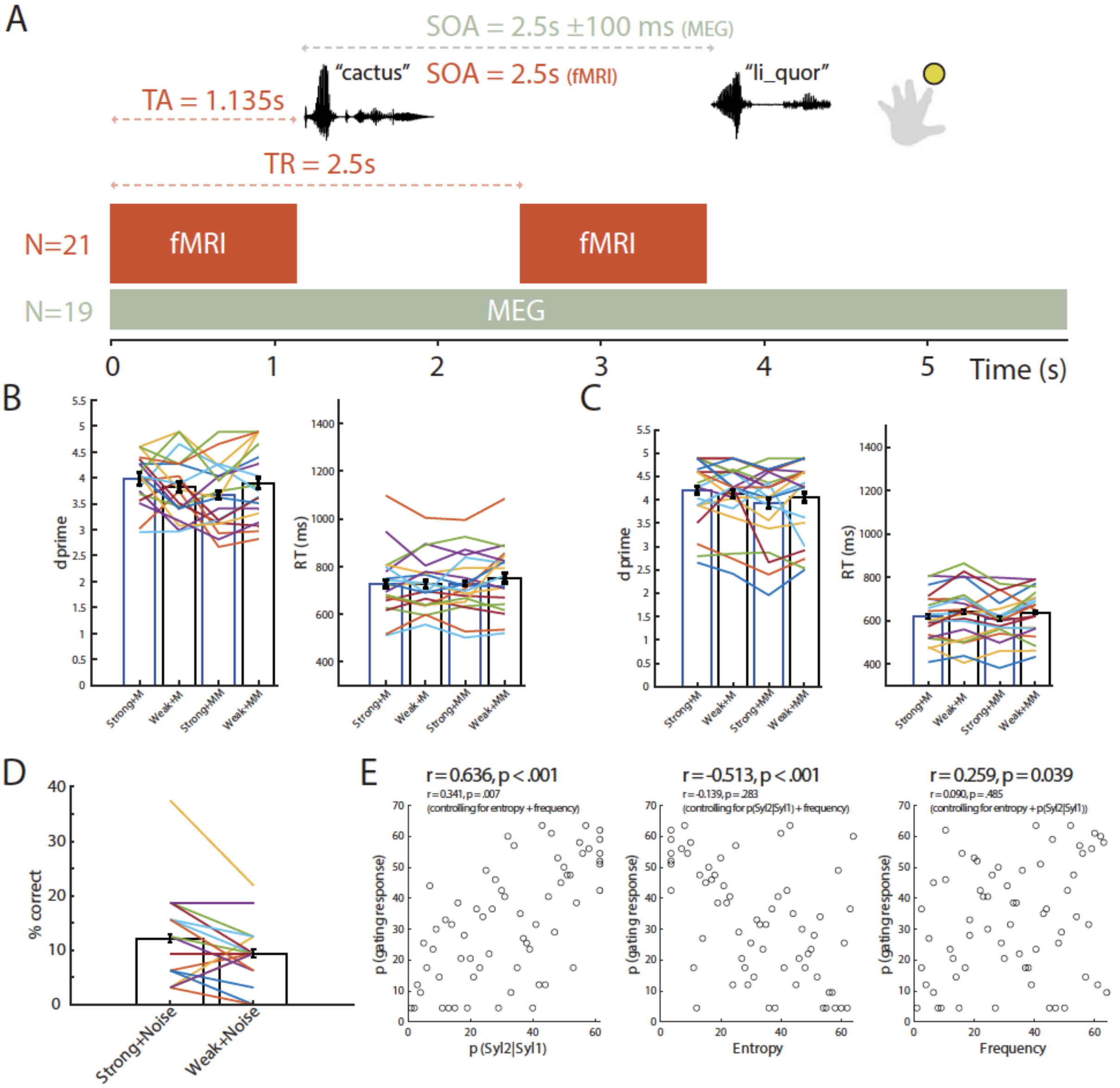

Figure S1- Method and behavioural results. (A) Participants listened to the spoken stimuli and detected a brief pause that appeared occasionally between Syl1 and Syl2 by responding with a button press. MEG and fMRI were recorded in two separate groups of participants (N = sample size for each group). Two example trials are indicated with stimulus and scan timings. TA = time of fMRI scan acquisition. TR = repetition time for fMRI scans. SOA = Stimulus Onset Asynchrony. s = seconds. ms = milliseconds. (B) Bar graph showing group-averaged d-primes and response times (target-present trials) for the pause detection task in the MEG experiment (with within-subject standard errors). Individual lines represent individual participants. (C) Same as panel B but for pause detection responses in the fMRI experiment. (D) Percentage correct accuracy of pseudoword memory in a cued recall test conducted after the fMRI scans. For this recall test, participants were presented with the stimuli after the second syllables were replaced with noise ('Strong+Noise' and 'Weak+Noise' items) and asked to recall previously heard pseudowords that started with the same first syllable. (E) Scatter plots of the proportion of participants identifying target items in a web-based gating pre-test study plotted against lexical statistics for p(Syl2|Syl1), entropy and word frequency (each circle representing one of the 64 word items used in the experiment). Correlation coefficients (and p values) for each relationship are shown above each graph with further partial correlations reported within brackets (all two-sided). Source data are provided as a Source Data file.

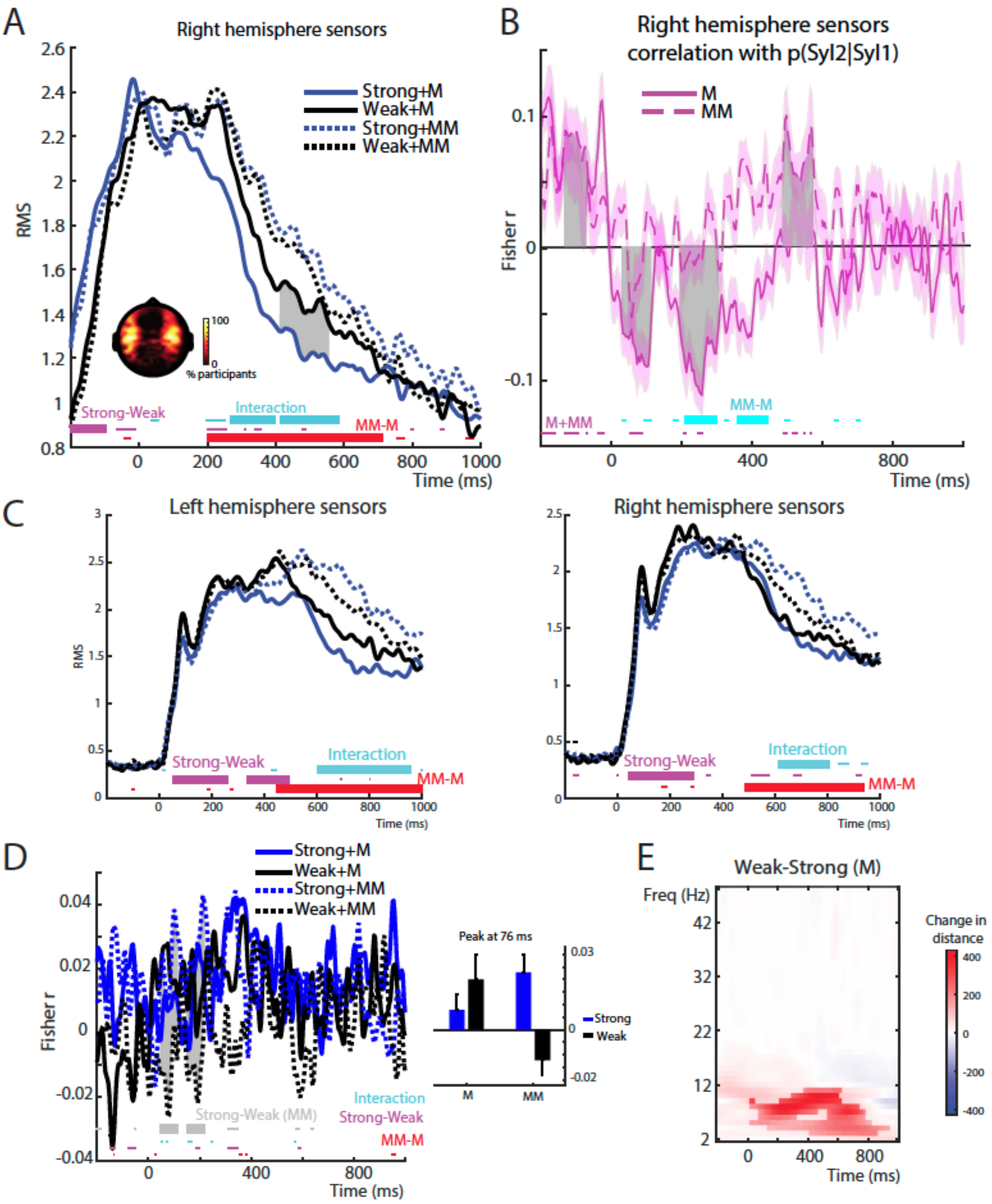

Figure S2- Supplementary MEG analyses. (A) Same as Figure 2A but for right hemisphere sensors. (B) Same as Figure 2B but for right hemisphere sensors. (C) Same as Figure 2A for MEG signal timelocked to Syl1 onset. (D) Group-averaged correlation between MEG pattern distances and Syl2 phonetic dissimilarities in each condition. Thin horizontal bars at the bottom of the graph indicate timepoints when main effects and the interaction were significant at an uncorrected p < .05 threshold based on cluster-permutation tests (no timepoints survived correction for multiple comparisons). Grey shaded areas indicate the two clusters during which there was a significant difference between Strong and Weak items in the Mismatch condition (p < .05 FWE clusterwise corrected). The inset bar plot shows group means for each condition (and within-subject standard errors as before) after averaging over the temporal extent of the first cluster (peaking at 76 ms). All tests are two-sided. (E) Pattern analysis showing simple effect of Weak - Strong (Match) on MEG pattern distances (p < .05 FWE clusterwise corrected; two-sided cluster-permutation test). Source data are provided as a Source Data file.

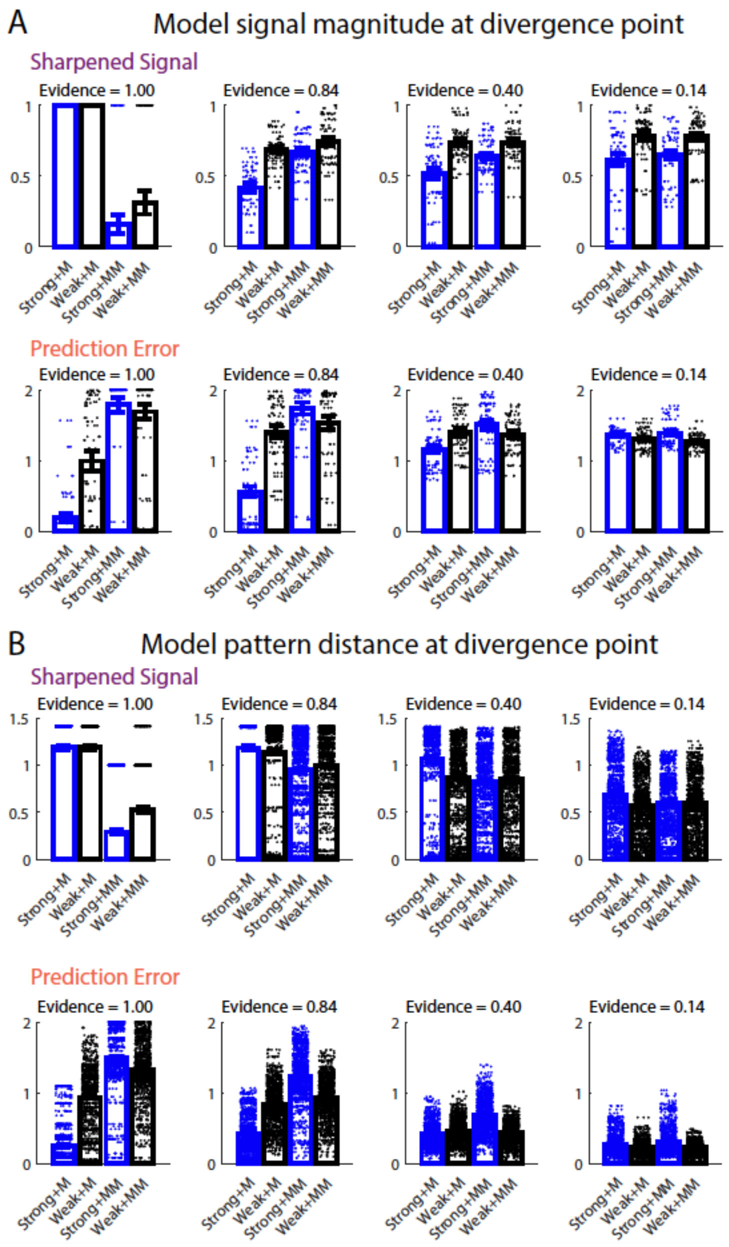

Figure S3- Supplementary computational modelling. Same as Figure 1D and 1E but showing the impact of varying sensory uncertainty levels (using the Softmax Temperature parameter) for Sharpened Signal (upper panels) simulations and Prediction Error (lower panels) for simulations of (A) Univariate signal magnitude and (B) Multivariate pattern distance results. For univariate simulations, sharpened signals were summarised as the sum of log activity over segment units while prediction errors were summarised as the sum of absolute activity. To give an indication of the degree of sensory uncertainty for each simulation, above each graph we show the average along the diagonal of the acoustic similarity matrix used for modelling the sensory evidence. This quantity can be interpreted as the average probability of correctly identifying speech segments. The simulation results depicted in Figure 1D and 1E correspond to the graphs in the second column (i.e. Evidence = 0.84).

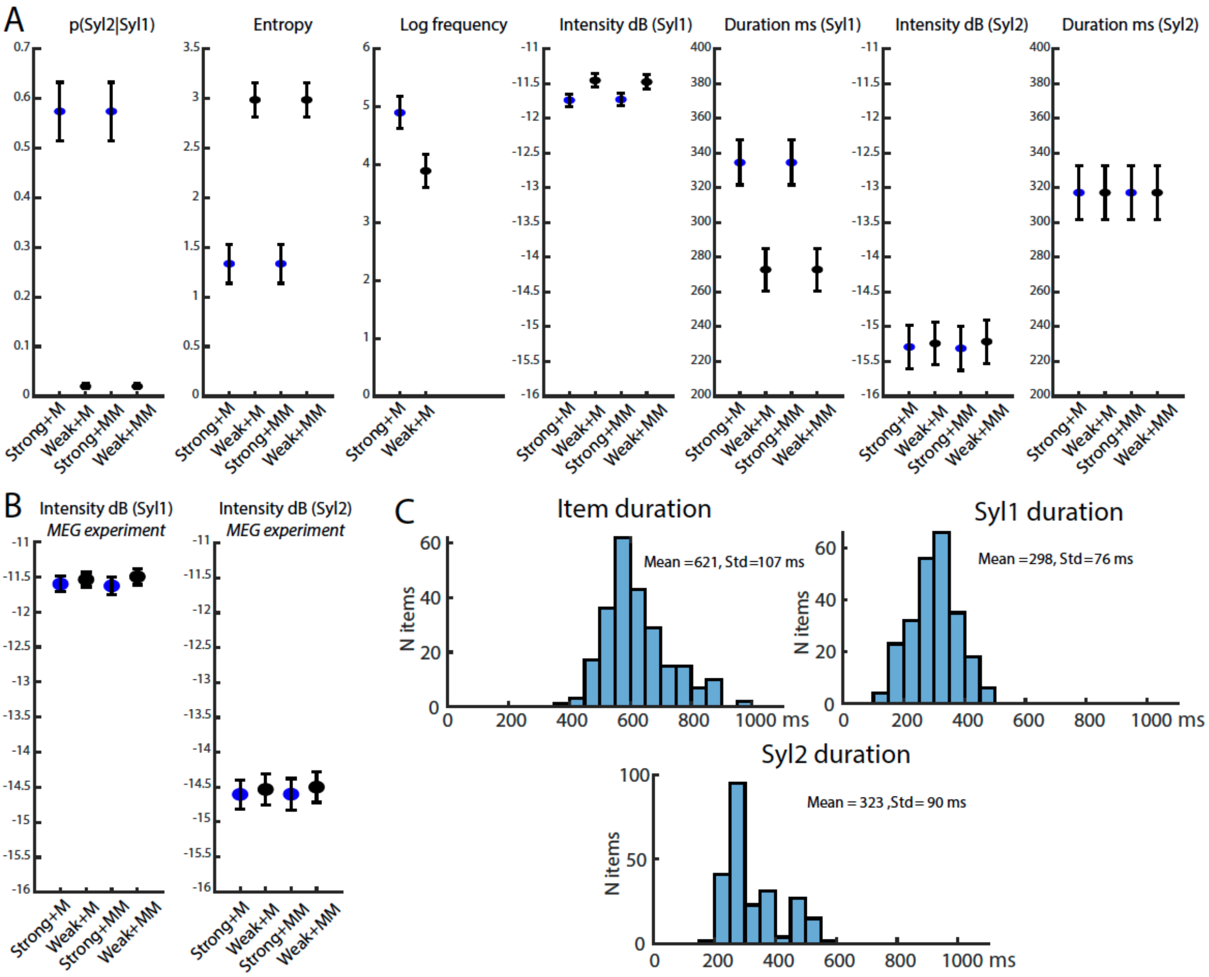

Figure S4- Stimulus properties. (A) Stimulus properties for the fMRI experiment (means and standard errors of the mean). (B) The RMS intensity of the stimuli for the MEG experiment, which were slightly different to those for the fMRI experiment (all other stimulus properties did not differ) (C) Histograms showing the distribution of item durations in our stimuli as well as Syl1 and Syl2 durations.

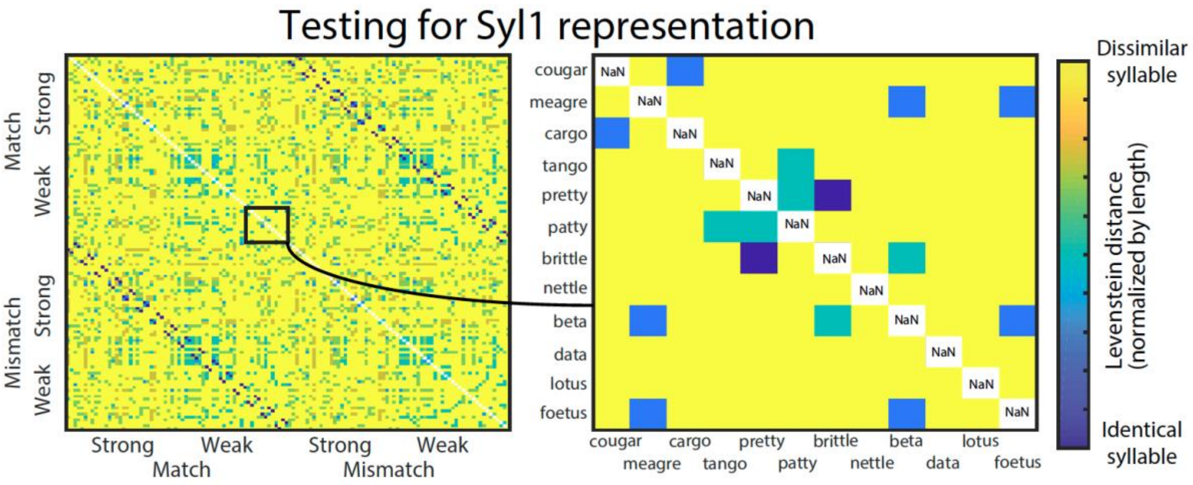

Figure S5- Phonetic dissimilarity matrix expressing the normalized Levenshtein distance between phonetic transcriptions of Syl1 in different items.

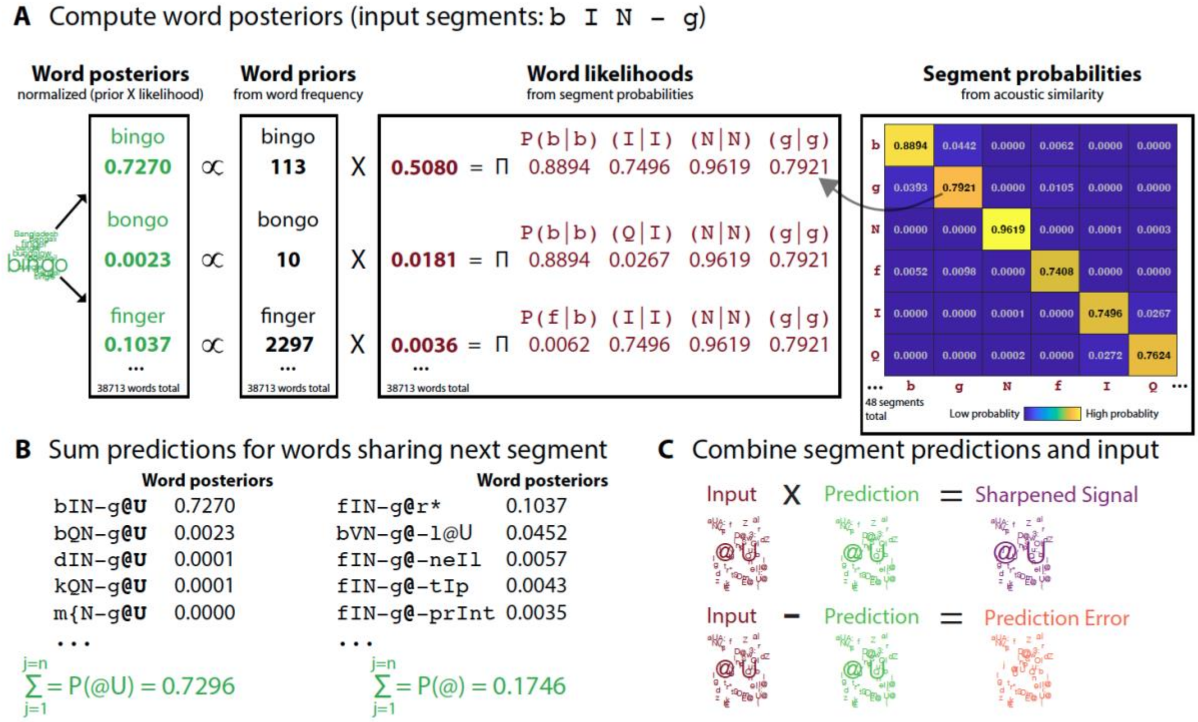

Figure S6- Illustration of each step in simulations for the example sequence of segments /b I N – g/ (i.e. after the first segment in the second syllable of "bingo"). A) The first step in the simulations is to compute the posterior probability of each word (shown in green for three selected words), by multiplying word priors (frequency of usage as shown, each of which is divided by the total word count for the corpus, 18585424) with word likelihoods (shown in brown) and normalising the result to sum to one. We compute word likelihoods as the product of segment probabilities, which are based on the acoustic similarity between segments. Selected segment probabilities are shown in the probability matrix in top right of figure. Each row in this matrix can be interpreted as the sensory evidence for different segments given an input segment (with yellow indicating strong evidence and blue indicating weak evidence). For example, given the input segment /b/, the sensory evidence for /b/ is high (P=0.8894), but also to some degree, there is evidence for acoustically similar segments /g/ (P=0.0442) and /f/ (P=0.0062). The non-zero probability of segment confusions is sufficient to make the likelihood of other, similar-sounding words non-zero (e.g. "finger"). B) In the second step, we compute predictions for each upcoming speech segment by summing posterior probabilities over words sharing the same next segment. Here we show posterior probabilities for two example segments /@U/ in "bingo", "bongo", etc, and /@/ in "finger", "bungalow", etc. Probabilities are similarly computed for each possible subsequent segment. C) In the final step, segment predictions (green distribution) are multiplied by individual segment probabilities (brown distribution) and normalized to sum to one to simulate the sharpened signal computation. To simulate the prediction error computation, segment predictions are instead subtracted from segment probabilities. The resulting distributions show how representations of expected segments (/@U/) are enhanced in sharpened signal computations (purple) and suppressed in prediction error computations (orange).
