# Supplementary material for: Convergent neural signatures of speech prediction error are a biological marker for spoken word recognition
Source: Nat Commun. 2024 Nov 18;15:9984. doi: 10.1038/s41467-024-53782-5 (PMC11574182; doi:10.1038/s41467-024-53782-5)
Supplement: Supplementary file 2 — Reporting Summary [file 41467_2024_53782_MOESM2_ESM.pdf]

nature portfolio

Corresponding author(s): Ediz Sohoglu

Last updated by author(s): 12/09/2024

Reporting Summary

Nature Portfolio wishes to improve the reproducibility of the work that we publish. This form provides structure for consistency and transparency in reporting. For further information on Nature Portfolio policies, see our Editorial Policies and the Editorial Policy Checklist.

Statistics

For all statistical analyses, confirm that the following items are present in the figure legend, table legend, main text, or Methods section.

n/a	Confirmed	
☐	☒	The exact sample size (n) for each experimental group/condition, given as a discrete number and unit of measurement
☐	☒	A statement on whether measurements were taken from distinct samples or whether the same sample was measured repeatedly
☐	☒	The statistical test(s) used AND whether they are one- or two-sided<br>*Only common tests should be described solely by name; describe more complex techniques in the Methods section.*
☐	☒	A description of all covariates tested
☐	☒	A description of any assumptions or corrections, such as tests of normality and adjustment for multiple comparisons
☐	☒	A full description of the statistical parameters including central tendency (e.g. means) or other basic estimates (e.g. regression coefficient) AND variation (e.g. standard deviation) or associated estimates of uncertainty (e.g. confidence intervals)
☐	☒	For null hypothesis testing, the test statistic (e.g. F, t, r) with confidence intervals, effect sizes, degrees of freedom and P value noted<br>*Give P values as exact values whenever suitable.*
☒	☐	For Bayesian analysis, information on the choice of priors and Markov chain Monte Carlo settings
☐	☒	For hierarchical and complex designs, identification of the appropriate level for tests and full reporting of outcomes
☐	☐	Estimates of effect sizes (e.g. Cohen's d, Pearson's r), indicating how they were calculated

Our web collection on statistics for biologists contains articles on many of the points above.

Software and code

Policy information about availability of computer code

Data collection	Psychtoolbox 3.0.14 in Matlab was used to control stimulus delivery and response collection.
Data analysis	MaxFilter 2.2, SPM12 (r7487 for MEG and r7219 for fMRI), Fieldtrip (as distributed with SPM12 r7487) and The Decoding v3.991 toolboxes in Matlab were used to analyse fMRI and MEG data, in conjunction with custom Matlab scripts. Custom Matlab code was used for the computational simulations. Code for analysis and simulations will be shared on OSF upon publication.<br><br>The code for the analyses and computational simulations in this study are available on OSF (https://osf.io/wjd4s/).

For manuscripts utilizing custom algorithms or software that are central to the research but not yet described in published literature, software must be made available to editors and reviewers. We strongly encourage code deposition in a community repository (e.g. GitHub). See the Nature Portfolio guidelines for submitting code & software for further information.

Data

Policy information about availability of data

All manuscripts must include a data availability statement. This statement should provide the following information, where applicable:

- Accession codes, unique identifiers, or web links for publicly available datasets
- A description of any restrictions on data availability
- For clinical datasets or third party data, please ensure that the statement adheres to our policy

The stimuli and data for this study are available on OSF (https://osf.io/wjd4s/).

Research involving human participants, their data, or biological material

Policy information about studies with human participants or human data. See also policy information about sex, gender (identity/presentation), and sexual orientation and race, ethnicity and racism.

Reporting on sex and gender	40 participants (19 in the MEG experiment, 21 in the fMRI experiment) were tested after being informed of the study's procedure, which was approved by the Cambridge Psychology Research Ethics Committee. All were right-handed, native speakers of English, aged between 18 and 40 years and had no self-reported history of hearing impairment or neurological disease. The mean age in the MEG experiment was 25 years (SD = 4.63; 12 female, 7 male) and 23 years in the fMRI experiment (SD = 3.14; 13 female, 8 male).
Reporting on race, ethnicity, or other socially relevant groupings	N/A
Population characteristics	N/A
Recruitment	We recruited from local participant databases. Recruitment criteria were: right-handed, native speakers of English, aged between 18 and 40 years and had no self-reported history of hearing impairment or neurological disease.
Ethics oversight	The study's procedures were approved by the Cambridge Psychology Research Ethics Committee

Note that full information on the approval of the study protocol must also be provided in the manuscript.

Field-specific reporting

Please select the one below that is the best fit for your research. If you are not sure, read the appropriate sections before making your selection.

[x] Life sciences [ ] Behavioural & social sciences [ ] Ecological, evolutionary & environmental sciences

For a reference copy of the document with all sections, see nature.com/documents/nr-reporting-summary-flat.pdf

Life sciences study design

All studies must disclose on these points even when the disclosure is negative.

Sample size	Because this study used new stimuli and a new paradigm, we did not have a priori estimates of effect size. Therefore the sample size for both experiments (MEG and fMRI) was based on that used in previous MEG and fMRI studies of speech processing.
Data exclusions	Data from single subjects (4 for the fMRI experiment; 5 for the MEG experiment) were excluded because of technical issues, data quality (excessive noise from head movements) or poor performance in the behavioral task (indicating lack of attention).<br>For the MEG experiment, epochs with large amplitude signals (indicative of artifacts) were discarded (time- and channel-averaged power larger than 2 standard deviations from the condition-specific mean for signal magnitude analysis; power larger than 3 standard deviations from the pooled mean for pattern analysis).
Replication	Because we recorded neural responses to the same stimuli in two separate groups of participants for the MEG and fMRI experiments, these can be considered an internal replication of the study's main results. No other replications have been conducted.
Randomization	We employed a within-subject design and randomized the order of trials within-subject. Participants were recruited from the volunteer research panel of the MRC Cognition and Brain Sciences Unit.
Blinding	This is not relevant to the current study because we used a within-subject design.

Reporting for specific materials, systems and methods

We require information from authors about some types of materials, experimental systems and methods used in many studies. Here, indicate whether each material, system or method listed is relevant to your study. If you are not sure if a list item applies to your research, read the appropriate section before selecting a response.

Materials & experimental systems

n/a	Involved in the study
☒	☐ Antibodies
☒	☐ Eukaryotic cell lines
☒	☐ Palaeontology and archaeology
☒	☐ Animals and other organisms
☒	☐ Clinical data
☒	☐ Dual use research of concern
☒	☐ Plants

Methods

n/a	Involved in the study
☒	☐ ChIP-seq
☒	☐ Flow cytometry
☐	☒ MRI-based neuroimaging

Plants

Seed stocks: N/A

Novel plant genotypes: N/A

Authentication: N/A

Magnetic resonance imaging

Experimental design

Design type: Event-related

Design specifications: On each trial, listeners heard one of the spoken items separated by a stimulus onset asynchrony (SOA) of 2.5 sec. For the fMRI experiment, a constant SOA was used, and stimuli were presented at the offset of the scans (sparse imaging sequence). Trials were randomly ordered during each of five presentation blocks of 288 trials. Each block consisted of a single presentation of all stimuli plus 'Pause' stimuli. Across all five blocks, the order of 'Pause' and 'No Pause' items was completely randomised; in total there was one 'Pause' item for every six 'No Pause' items, with this ratio constant across stimulus type (Strong+Match, Weak+Match etc.).

Behavioral performance measures: To maintain listeners' attention, participants were asked to detect brief (200 ms) pauses inserted between the syllables of the spoken items in occasional target items.

Acquisition

Imaging type(s): Functional and structural images were acquired.

Field strength: 3T

Sequence & imaging parameters: Imaging data were collected on a Siemens 3 Tesla Prisma MRI scanner (http://www.siemens.com). A total of 291 echo planar imaging (EPI) volumes were acquired in each of 5 scanning runs, using a 32-channel head coil and a multiband sparse imaging sequence (TR = 2.50 sec; TA = 1.135 sec; TE = 30 ms; 48 slices covering the whole brain; flip angle = 78 deg; in-plane resolution = 3 × 3 mm; matrix size = 64 × 64; echo spacing = 0.5 ms; inter-slice gap = 25%). After the third run, field maps were acquired (short TE = 10 ms; long TE = 12.46 ms). The experimental session commenced with the acquisition of a high-resolution T1-weighted structural MRI scan (TR = 2250 ms; TE = 2.99 ms; flip angle = 9°; 1 mm isotropic resolution; matrix size: 256 × 240 × 192 mm; GRAPPA acceleration factor PE = 2; Reference lines PE = 24).

Area of acquisition: Whole brain

Diffusion MRI: ☐ Used ☒ Not used

Preprocessing

Preprocessing software: SPM12

Normalization: Realigned images were co-registered to the mean functional image and then subjected to statistical analysis. For signal magnitude analysis, prior to further processing, images were also normalized to the Montreal Neurological Institute (MNI) template image using the parameters from the segmentation of the structural image (resampled resolution: 2 × 2 × 2 mm) and smoothed with a Gaussian kernel of 6 mm full-width at half-maximum. For pattern analysis, normalization and smoothing

	were performed only after computing pattern distances.
Normalization template	MNI template brain included in SPM12
Noise and artifact removal	After discarding the first four volumes to allow for magnetic saturation effects, the remaining images were realigned and unwarped to the first volume to correct for movement of participants during scanning. Also at the unwarping stage, the acquired field maps were used to correct for geometric distortions in the EPI due to magnetic field variations.
Volume censoring	N/A

Statistical modeling & inference

Model type and settings	Analyses included univariate and multivariate (neural pattern distances) procedures. All analyses were based on random effects inference.
Effect(s) tested	We tested for main effects of prediction strength and congruency, and their interaction, using ANOVA. Graded analyses were based on Spearman correlations between prediction strength and neural data (signal magnitude or pattern distances), in conjunction with t-tests for testing between-differences in correlations.

Specify type of analysis: ☐ Whole brain ☐ ROI-based ☒ Both

Anatomical location(s): ROIs were functionally defined based on statistically independent contrasts.

Statistic type for inference (See Eklund et al. 2016)	Inference was conducted parametrically for fMRI analysis, controlling the family-wise error (FWE) rate using random field theory.
Correction	Reported effects were obtained by using a cluster defining height threshold of $p < .001$ with a cluster extent threshold of $p < .05$ (FWE corrected), unless otherwise stated.

Models & analysis

n/a	Involved in the study
☒	☐ Functional and/or effective connectivity
☒	☐ Graph analysis
☒	☐ Multivariate modeling or predictive analysis
